# Colorectal Cancer Identification Methods Among Kansas Medicare Beneficiaries, 2008–2010

**DOI:** 10.5888/pcd12.140543

**Published:** 2015-07-09

**Authors:** Sue-Min Lai, Jessica Jungk, Sarma Garimella

**Affiliations:** Author Affiliations: Jessica Jungk, Sarma Garimella, Kansas Cancer Registry, Department of Preventive Medicine and Public Health, University of Kansas Medical Center, Kansas City, Kansas.

## Abstract

**Introduction:**

Population-based data are limited on how often colorectal cancer (CRC) is identified through screening or surveillance in asymptomatic patients versus diagnostic workup for symptoms. We developed a process for assessing CRC identification methods among Medicare-linked CRC cases from a population-based cancer registry to assess identification methods (screening/surveillance or diagnostic) among Kansas Medicare beneficiaries.

**Methods:**

New CRC cases diagnosed from 2008 through 2010 were identified from the Kansas Cancer Registry and matched to Medicare enrollment and claims files. CRC cases were classified as diagnostic-identified versus screening/surveillance-identified using a claims-based algorithm for determining CRC test indication. Factors associated with screening/surveillance-identified CRC were analyzed using logistic regression.

**Results:**

Nineteen percent of CRC cases among Kansas Medicare beneficiaries were screening/surveillance-identified while 81% were diagnostic-identified. Younger age at diagnosis (65 to 74 years) was the only factor associated with having screening/surveillance-identified CRC in multivariable analysis. No association between rural/urban residence and identification method was noted.

**Conclusion:**

Combining administrative claims data with population-based registry records can offer novel insights into patterns of CRC test use and identification methods among people diagnosed with CRC. These techniques could also be extended to other screen-detectable cancers.

## Introduction

Colorectal cancer (CRC) is the third most common cancer and third leading cause of cancer death in Kansas (1). In 2002, the US Preventive Services Task Force (USPSTF) strongly recommended that people aged 50 years or older be screened for CRC on the basis of evidence that screening is effective in reducing CRC mortality rates (2,3). As a result of this recommendation, CRC screening has been widely promoted by many groups, including the National Cancer Institute, the Centers for Disease Control and Prevention (CDC), and the American Cancer Society (ACS). Use of CRC tests among US adults has been increasing (4–6). From 2000 to 2010, the percentage of US adults aged 50 to 75 receiving any CRC screening test within recommended intervals increased from 38.6% to 59.1% (4,7). At the same time, CRC incidence has been declining and the proportion of cases diagnosed at a localized stage has been increasing, trends attributed to a combination of risk-factor reduction and increased screening rates (8). However, CRC screening rates still lag behind those for other effective cancer screening tests. In 2010, less than half of CRC cases were diagnosed at a localized stage in both Kansas (41%) and the United States (39%) (1,7,9). Although CRC screening is promoted as a key tool for improving CRC outcomes, few data are available on how often CRC cases are identified through screening or surveillance in asymptomatic patients versus diagnostic workup for symptoms, particularly at the population level. Documenting trends in CRC identification methods could provide additional insight into the contributions of screening to CRC prevention and morbidity reduction. In addition, analyses of patient and tumor characteristics by identification method could identify population subgroups not benefiting from CRC screening and tumor subgroups not amenable to identification by screening. We sought to explore the circumstances leading to the identification of CRC, to examine relationships between identification method and patient and tumor characteristics, and to develop a process for assessing CRC identification methods among Medicare-linked CRC cases from a population-based cancer registry.

## Methods

### Study population and data sources

This study was approved by the University of Kansas Medical Center Institutional Review Board. Kansas residents aged 65 years or older who were diagnosed with invasive CRC from 2008 through 2010 were identified from the population-based Kansas Cancer Registry (n = 2,497) and matched to Medicare enrollment files using Social Security numbers, with further confirmation based on date of birth, sex, race, and residence zip code. A deterministic matching approach was used for the linkage. A total of 2,378 patients (95.2%) were successfully matched between the 2 databases. CRC cases were defined according to the Surveillance, Epidemiology, and End Results (SEER) Site Recode values for the Colon (21041–21049, 21051) and Rectum (21052). Only patients continuously enrolled in both Medicare Parts A and B (fee-for-service) with no health maintenance organization coverage during the year before diagnosis were included. CRC-related diagnoses and procedures were identified using Part A inpatient hospital and skilled nursing facility claims (MedPAR file), Part A institutional outpatient provider claims (Outpatient file) and Part B noninstitutional physician/supplier claims (Carrier file). For patients with multiple CRC primaries diagnosed from 2008 through 2010, the first incident case was selected for analysis. Patients were excluded if they had a prior invasive CRC (n = 120), were missing month or day of diagnosis or both (n = 24), were ascertained by the registry solely on the basis of a death certificate (n = 34), were eligible for Medicare because of disability or end-stage renal disease (n = 166), were not continuously enrolled in Medicare Part A and Part B in the year before diagnosis (n = 375), or had minimal or no claims history around their diagnosis date (n = 70). The final study population was 1,589 patients.

### Assigning CRC identification method

CRC identification method (screening/surveillance vs diagnostic) was determined by evaluating the indication for use of any CRC-related tests (colonoscopy, computed tomography [CT] colonography, double-contrast barium enema, fecal occult blood test [FOBT]/fecal immunochemical test [FIT], sigmoidoscopy) documented in Medicare claims during the 60 days before diagnosis. Because CRC screening and surveillance are test-based, patients without a CRC-related test in the 60-day window were assumed to be diagnostic-identified (n = 256). Current Procedural Terminology (CPT), Healthcare Common Procedure Coding System (HCPCS), and *International Classification of Diseases, Ninth Revision, Clinical Modification* (ICD-9-CM) procedure codes were used to identify CRC-related tests on claims from physician encounters, hospital outpatient encounters, and inpatient admissions (Table 1). For patients who had a colonoscopy claim in the 60-day window (n = 1,257), the nonparametric classification and regression tree (CART) algorithm for 2-level colonoscopy indication (diagnostic vs average-risk screening/high-risk screening/surveillance) developed by Ko et al was applied to determine colonoscopy indication and corresponding CRC identification method (10). The CART algorithm uses ICD-9-CM diagnosis codes from the colonoscopy claim and claims during the year before to determine indication and does not rely on specific colonoscopy procedure codes. The sensitivity and specificity for this algorithm were reported as 77% and 90%, respectively, by the authors (10). For patients who did not have a colonoscopy in the 60-day window but did have another CRC-related test (CT colonography, double-contrast barium enema, FOBT/FIT, sigmoidoscopy) (n = 294), claims from the year before the test date were examined for CRC symptom ICD-9-CM diagnosis codes (Table 1). Among this group, patients with 1 or more claims with a CRC symptom diagnosis code were classified as diagnostic-identified. The Figure summarizes the CRC identification classification results for all patients.

**Table 1:** Procedure Codes Used to Identify CRC Tests and Diagnosis Codes Used to Identify CRC Symptoms in Medicare Claims

Tests and Symptoms	CPT Procedure	HCPCS Procedure	ICD-9-CM Procedure	ICD-9-CM Diagnosis
**CRC tests**				
Colonoscopy	44388–44394, 44397, 45355, 45378–45387, 45391, 45392	G0105, G0121	45.21–45.23, 45.25, 45.41–45.43, 48.36	—
CT colonography	0066T, 0067T, 74261–74263	—	—	—
Double-contrast barium enema	74270, 74280	G0106, G0120, G0122	—	—
FOBT/FIT	82270–82274	G0107, G0328	—	—
Sigmoidoscopy	45300, 45303, 45305, 45307–45309, 45315, 45317, 45320, 45321, 45327, 45330–45342, 45345	G0104	45.24, 48.21–48.25	—
**CRC symptoms**				
Abdominal mass	—	—	—	789.30–789.39
Abdominal/rectal pain	—	—	—	569.42, 789.00–789.07, 789.09
Anemia (iron deficiency and NOS)	—	—	—	280.0, 280.9, 285.1, 285.9
Change in bowel habits	—	—	—	787.99
Constipation	—	—	—	564.00–564.02, 564.09
Diarrhea	—	—	—	787.91
Enteritis and colitis	—	—	—	555.1, 555.2, 555.9, 556.0–556.5, 556.8– 557.1, 557.9, 558.1–558.4
Gastrointestinal bleeding	—	—	—	569.3, 578.1, 578.9, 792.1
Weight loss	—	—	—	783.21

Abbreviations: —, not applicable, CRC, colorectal cancer; CPT, Current Procedural Terminology; CT, computed tomography; FOBT, fecal occult blood test; FIT, fecal immunochemical test; HCPCS, Healthcare Common Procedure Coding System; ICD-9-CM, International Classification of Diseases, Ninth Revision, Clinical Modification; NOS, not otherwise specified.

**Figure:**
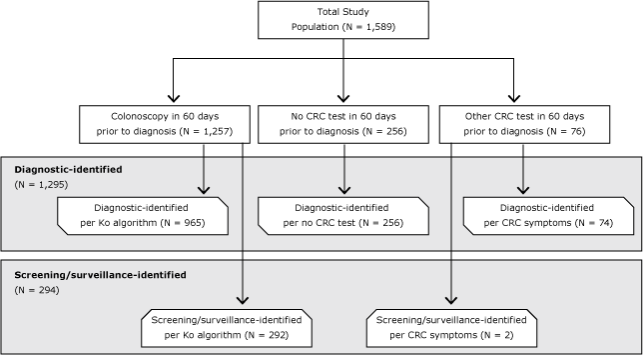
Identification method classification process and results for invasive colorectal cancer (CRC), Kansas Medicare beneficiaries, 2008–2010. “Ko algorithm” refers to classification and regression tree algorithm for colonoscopy indication (diagnostic vs average-risk screening/high-risk screening/surveillance) developed by Ko et al (10).

### Statistical analyses

Independent variables included age, sex, race, marital status, rural/urban residence and anatomic site from Kansas Cancer Registry (KCR) records along with comorbidity score and income level from Medicare records. A comorbidity score was calculated using the Charlson comorbidity index as adapted for physician and hospital administrative claims data by the National Cancer Institute and presented on the SEER-Medicare website (11,12). MedPAR, Outpatient and Carrier claims from the year preceding diagnosis were used to determine the comorbidity score. Claims from the 30 days before diagnosis were excluded to avoid identifying complications or comorbidities resulting from the cancer diagnosis. Patients were categorized as low-income or not low-income based on their eligibility for Medicare premium subsidy (full or partial Medicaid dual-eligibility or Part D Low-Income Subsidy or both) in any month during the year before diagnosis. Rural/urban residence was defined using Rural Urban Commuting Area (RUCA) zip code approximation codes for the patient’s zip code at diagnosis (13). Stage at diagnosis was classified by the SEER Summary Stage 2000 system as localized, regional, distant, and unstaged. Associations between categorical variables and CRC identification method were examined using Pearson χ^2^ tests. Bivariate and multiple logistic regressions were used to calculate unadjusted and adjusted odds ratios (ORs) and 95% confidence intervals (CIs) for factors associated with CRC identification through screening/surveillance. All significance tests were 2-sided at the .05 level. Only independent variables with a *P* value of <.15 in the bivariate logistic regression analysis were included in the multiple logistic regression analysis. SAS version 9.3 for Windows was used for all analyses.

## Results

A total of 1,589 CRC cases who met the study eligibility criteria were included in the analysis. Of these, 1,333 patients had a claim for any CRC test in the 60 days before diagnosis. The distribution of CRC test use included 1,257 with colonoscopy, 176 with FOBT/FIT, 140 with sigmoidoscopy, 40 with double-contrast barium enema and 2 with CT colonography. There were 267 patients who had claims for more than 1 type of CRC test in the 60 days before diagnosis. Nineteen percent (n = 294) of the patients were classified as having screening- or surveillance-identified CRC (Table 2). Patients with screening/surveillance-identified CRC were more likely to be diagnosed at a local stage (*P* < .001), aged 65 to 74 years (*P* < .001), and married (*P* = .003). Patients with a colonoscopy claim were more likely to be classified as having screening- or surveillance-identified CRC than patients with claims for another CRC test (23% vs 3%, *P* < .001).

**Table 2:** Characteristics of Kansas Medicare Beneficiaries Diagnosed With Invasive Colorectal Cancer by Identification Method, 2008–2010

Demographic Variables	Identification Method, N (%)			*P* Value^c^
	All (n = 1,589, 100%)	Screening/Surveillance-identified (n = 294, 19%)	Diagnostic-identified (n = 1,295, 81%)	
Diagnosis year				
2008	554 (35)	104 (35)	450 (35)	.57
2009	531 (33)	91 (31)	440 (34)	
2010	504 (32)	99 (34)	405 (31)	
Age at diagnosis (years)				
65–74	529 (33)	138 (47)	391 (30)	<.001
75–84	692 (44)	123 (42)	569 (44)	
≥85	368 (23)	33 (11)	335 (26)	
Sex				
Male	717 (45)	148 (50)	569 (44)	.05
Female	872 (55)	146 (50)	726 (56)	
Race				
White/Other	1,538 (97)	287 (98)	1,251 (97)	.37
Black	51 (3)	7 (2)	44 (3)	
Marital status^a^				
Married	775 (51)	162 (60)	613 (50)	.003
Not married	733 (49)	110 (40)	623 (50)	
Charlson comorbidity index				
0	789 (50)	162 (55)	627 (48)	.10
1–2	631 (40)	107 (36)	524 (40)	
≥3	169 (11)	25 (9)	144 (11)	
Income				
Low-income	175 (11)	23 (8)	152 (12)	.05
Not low-income	1,414 (89)	271 (92)	1,143 (88)	
Rural/urban residence^b^				
Urban	673 (42)	137 (47)	536 (41)	.35
Large rural	385 (24)	66 (22)	319 (25)	
Small rural	201 (13)	31 (11)	170 (13)	
Isolated	329 (21)	60 (20)	269 (21)	
Stage at diagnosis				
Local	739 (47)	181 (62)	558 (43)	<.001
Regional	507 (32)	78 (27)	429 (33)	
Distant	247 (16)	18 (6)	229 (18)	
Unstaged	96 (6)	17 (6)	79 (6)	
Anatomic site				
Colon	1,363 (86)	257 (87)	1,106 (85)	.37
Rectum	226 (14)	37 (13)	189 (15)	

a Missing marital status (n = 81).

b Missing rural/urban residence (n = 1).

c *P* values calculated using Pearson χ^2^ test.

In the bivariate analysis, younger age, male sex, and being married were significantly associated with increased odds of having screening/surveillance-identified CRC (Table 3). Low-income patients were significantly less likely to have screening/surveillance-identified CRC (*P* = .05). In the multiple logistic regression analysis, only being aged 65 to 74 years remained a significant predictor of having screening- or surveillance-identified CRC. Compared with this age group, patients aged 75 to 84 years had 41% lower odds of having screening- or surveillance-identified CRC; patients aged 85 years or older (*P* < .001) had 72% lower odds.

**Table 3:** Predictors of Screening/Surveillance-Identified Colorectal Cancer Among Kansas Medicare Beneficiaries, 2008–2010

Demographic Factor	Unadjusted OR (95% CI)^c^	*P *Value	Adjusted OR (95% CI)^c^	*P *Value
**Age at diagnosis, y**				
65–74	1 [Reference]	<.001	1 [Reference]	<.001
75–84	0.61 (0.47–0.81)		0.59 (0.45–0.79)	
>85	0.28 (0.19–0.42)		0.28 (0.18–0.44)	
**Sex**				
Female	1 [Reference]	.05	1 [Reference]	.72
Male	1.29 (1.00–1.67)		1.05 (0.79–1.40)	
**Race**				
White/Other	1 [Reference]	.38	NA	
Black	0.69 (0.31–1.56)			
**Marital status^a^**				
Not married	1 [Reference]	.003	1 [Reference]	.37
Married	1.50 (1.15–1.95)		1.15 (0.85–1.55)	
**Charlson comorbidity index**				
0	1 [Reference]	.10	1 [Reference]	.41
1–2	0.79 (0.60–1.04)		0.84 (0.63–1.12)	
>3	0.67 (0.42–1.06)		0.81 (0.50–1.31)	
**Income**				
Low-income	1 [Reference]	.05	1 [Reference]	.12
Not low-income	1.57 (0.99–2.48)		1.49 (0.91–2.44)	
**Rural/urban residence^b^**				
Urban	1 [Reference]	.35	NA	
Large rural	0.81 (0.59–1.12))			
Small rural	0.71 (0.47–1.10)			
Isolated	0.87 (0.62–1.22)			
**Anatomic site**				
Colon	1 [Reference]	.37	NA	
Rectum	0.84 (0.58–1.23)			

Abbreviations: CI, confidence interval; NA, factors were not included in adjusted odds ratio analysis; OR, odds ratio.

a Missing marital status (n = 81).

b Missing rural/urban residence (n = 1).

c *P* values calculated using Wald χ^2^ test.

## Discussion

In this study we developed a process for assessing CRC identification methods among Medicare-linked CRC cases from a population-based cancer registry by expanding on a previously published algorithm for determining colonoscopy indication. To maximize the number of CRC cases eligible for identification method classification, we also considered use of other CRC tests and symptom history among those without colonoscopy claims. Using our method, 19% of the Medicare-linked CRC cases diagnosed in 2008–2010 in Kansas were identified through screening or surveillance, while 81% were diagnostic-identified. Our finding of 19% screening/surveillance-identified in a population-based CRC cohort is consistent with that reported in a CRC cohort served by a single major medical institution (20%) during 2004–2011 (14). Another study in a health care system and a community hospital during 2002–2004 found that 9% of CRC cases were screening-identified (15). 

Certain factors could contribute to misclassification of identification method for CRC cases. First, it is possible that CRC test procedure codes and CRC-related symptom diagnosis codes for some patients were not documented in their Medicare claims history, either because of coding errors or because claims were sent to another payer (16–19). To minimize the impact of patients with non-Medicare primary payers before CRC diagnosis, we used the primary payer fields in the KCR database to identify patients with other sources of insurance and manually reviewed the completeness of their claims history. Of the patients included in the study, only 21 cases (1%) had a sole non-Medicare primary payer in the KCR database. Second, the 60-day window before diagnosis that we chose may not have captured all CRC-related tests. Sixteen percent of the patients (n = 256) in our study did not have a claim for a CRC test in the 60 days before diagnosis and as a result were classified as diagnostic-identified. This apparent lack of CRC testing could be legitimate because the patient was diagnosed through other means (eg, diagnostic imaging) or could be an artifact introduced by inaccurate diagnosis dates. We further examined the claims and KCR records for these 256 patients and found that 228 patients had a claim with a CRC-related symptom code within a year before diagnosis and an additional 15 patients had symptoms documented in the KCR database. Patients without CRC tests were more likely than their counterpart patients with CRC tests to have a CPT code documented for a diagnostic radiology procedure of the abdomen or pelvis (X-ray, CT, or MRI) in the 60 days before diagnosis. These insights increase our level of confidence in classifying these 256 patients as diagnostic-identified.

We found that CRC patients aged 65 to 74 years and who were male and married had increased odds of having screening/surveillance-identified CRC compared with their counterparts at the time of CRC diagnosis. However, younger age at diagnosis was the only factor associated with having screening/surveillance-identified CRC after adjusting for sex, marital status, comorbidity index, and income. Although low income had a marginal association with increased odds of having diagnostic-identified CRC, the significance level was diminished after adjusting for other risk factors. 

The observed decrease in screening/surveillance-identified CRC with increasing age could be due to several factors. Changes in CRC screening recommendations and Medicare coverage for CRC screening have occurred since 1998 (2,20). The 2002 USPSTF recommendation applied to all adults aged 50 or older without regard to an age at which to stop screening, but in 2008 the USPSTF recommended routine cancer screening to begin at age 50 and continue only until age 75 (in people with adequate screening histories). It is reasonable to assume that these changes affected various age cohorts differently; older patients would receive less lifetime benefit from recent improvements in screening technology and availability. We did not have access to enough claims history to evaluate long-term adherence to CRC screening recommendations for the patients in our study but historically low CRC screening rates indicate that many of the patients in the older age groups had an adequate history of negative screenings before their CRC diagnosis. Independent of screening recommendations, physicians may be less likely to recommend CRC screening in older people because of significant comorbidities, limited life expectancy, or both (21–23).

Understanding CRC screening patterns is important for CRC prevention and control at a population-based level. Unfortunately, estimating population-level use of CRC screening and the extent to which it contributes to CRC identification remains challenging, because no single data set is readily available to address this question. Various data sources including Medicare and Medicaid claims files, medical records, and surveys have been used to address this question but each has its own limitations and unique issues. Still, no single source exists for reliable information about how frequently CRC is identified through screening versus diagnostic workup. To examine CRC identification methods, our study not only used Medicare claims files to identify CRC tests but also considered CRC-related symptoms documented in the Medicare claims files as well as data in the Kansas Cancer Registry. 

As with any evaluation of clinical practice that relies on administrative claims data, a limitation of our study is the generalizability of our findings. The clinical experiences of the Medicare population and the way these experiences are reflected in their claims history may not be generalizable to other groups. However, despite this limitation, we believe that the combination of administrative claims data with population-based registry records can offer novel and important insights into screening patterns and identification methods among people diagnosed with CRC and that these techniques could also be extended to other screen-detectable cancers.
